# Fragmentary Clippings

**Published:** 1874-04

**Authors:** S. P. Cutler

**Affiliations:** Memphis, Tenn.


### ARTICLE II.

### *Fragmentary Clippings.*

BY S. P. CUTLER, M. D., D. D. S., MEMPHIS, TENN.

*Dental Cosmos*, *July*, 1873.—In Periscope, page 373, writer says: "After using arsenic and creasote, syringe the cavity well with pepsin and shut it in for two days.

I would simply ask for information, (as the writer did not say,) whether he left the arsenicized and pepsinized pulp in the tooth when the final filling is made? I would also ask if it would not be better to remove all of the arsenicized pulp possible before using the pepsin?

Writer speaking of the treatment of pulps on page 276, says: "If nothing but creasote is placed upon the pulp,
I was not aware of that fact; *i. e.*, that creasote would
it will in time run into decomposition."
cause decomposition of living tissue *per se* of any kind. I am seeking information. Further he says: "One of the great dangers of external wounds is in the exposure to the atmosphere; when exposed even for a short time, the germs of infusoria find a lodgment and produce suppuration. I do not say that it is necessary to be exposed to the air to produce suppuration, but exposure certainly hastens it. In putrefaction, animal and vegetable organisms invariably appeared."

A simple wound and putrefaction are somewhat different processes. I know full well that the idea of germs as the cause of suppurating and other morbid processes, is entertained by many writers; yet none of them have demonstrated or proven the fact beyond the simple coincidence of their presence in some instances, and in a very great many other instances to my certain knowledge, there was nothing of the kind, even in cases of fractured jaws, where there was a chronic discharge from the fractured bone direct; none, if such germs or organisms are found, as claimed by many; they undoubtedly are an accidental occurrence, and not a *causus vera* in the case, or a genesis of the morbid processes. In fact, these germs are rather a new or recent hobby not yet rode to death.

On the same page, writer, speaking of transplanting teeth, says: "Teeth have been transplanted, and yet their vitality has been retained." I very well know that the idea is prevalent with the profession, that transplanted teeth do resume their normal vitality; which fact I am not yet convinced of. After there has been perfect or absolute solution of continuity of a part so highly endowed with blood vessels and nerves as a tooth, and then a re-union of those vessels and nerves, perfect restoration of vitality, seems to me to be beyond belief. It has been demonstrated that patches of skin may be ingrafted on to old sores, and there grow and heal, and when a slice is cut from a finger or toe, and placed back and bound on fast, such slice may unite and grow fast, but as a rule, there is a loss of sensibility in such part, so I have been several times told. Now that the peridentium or periosteum may again form a union more or less perfect when a tooth has been transplanted, I am not yet prepared to deny; as in cases of devitalized teeth, the membrane retains a low degree of vitality, oftentimes in a pathological condition in both cases, in others comparatively healthy. This membrane is of low vitality, mostly connective tissue. Now what I wish to come at, is this: Has there ever been a case where a tooth has been completely dislodged from the socket, then replaced, and afterward a live and healthy pulp within? This is the point I want information upon. You have read of cases where sensibility in the dentine was found or supposed to be present; still, I have never been satisfied with any of these statements. I want to know if any dentist has ever opened the pulp cavity in any such tooth, months after the occurrence, and there found a live vascular pulp? That is the direct point we want more light and knowledge upon.

*Missouri Dental Journal*, July, 1873, p. 266, a writer on amalgam "thinks the tendency of amalgam fillings is towards spherical attraction, or to assume the globular form, consequently leaving the edges, more especially the angles, thereby causing leakage around the fillings by capillary attraction."

He speaks of burnishing before the filling is hard, so as to close up all places where shrinkage has taken place. This spherical attraction, which is a universal law of fluids when in the form of drops, so small that terrestrial attraction does not prevent, also equally applies to masses of matter in a plastic state throughout the universe, as we see modified in the form of all the bodies of the solar system.

The writer's experience and mine have been somewhat different on the subject of amalgam. He certainly meets with difficulties that I do not meet with as a rule, only in exceptional cases, many resulting from imperfect manipulation and bad material. I confess, I have not observed this spherical tendency to any great extent either in or out of the mouth, where more or less is left in a lump or ball after filling a tooth. That an amalgam filling, where the cavity has been properly prepared, with suitable retaining points, and the amalgam properly introduced, shrinks so as to cause leakage, I am prepared to speak in the negative; even with the old fashioned amalgam made of silver coin filed up and mixed with mercury, and plastered into the cavity with no instrument but the finger, where the cavity could be reached. I have seen such fillings, and know of some that are now in teeth of 25 years or more standing, even in what were considered bad teeth, and past filling with gold at the time; many dentists in this country can testify to the same fact. With the best amalgams in use, with our improved methods of insertion, I positively know that good and reliable fillings can and are duly inserted throughout this entire country. We often meet with fillings that appear to have started from their beds and be leaking at their sides; in such cases we further discover the filling projecting beyond the margins of the cavity. In such cases when sufficient time has elapsed, on removal of such filling, a dark coating is seen over the surface of the plug. This dark surface on analysis is found to be argentic oxide, insoluble in the fluids of the mouth, hence retained on the surface of the filling. Now where there is a defective filling suffering leakage, this oxide is formed, which occupies more space than the filling before such oxidation, and an expansive force exerted lifting the filling out of its bed, and frequently in such cases the cavity is found to be sound and firm, unless too long a time has elapsed.

Some may deny the above statements, but the proof is what I want. Any burnishing after the amalgam has set before complete hardening, I believe to be injurious to the filling, having a tendency to break up the crystaline process before completed.

*Dental Architecture.*—In the same journal there is a plan of a dental office and residence given, which is just the thing, and would fill the bill exactly. I wish I had just such a one, and sigh that I *havn't.* Memory naturally reverts back to the time when I first commenced the practice of dentistry; when instead of our patients coming to us as they now do, we went to them, and *for them*, and if entirely convenient for our patient to have the work done, we proceeded to business; if not, she would send us in word that it was not convenient to have her teeth operated upon that morning, and excuse her and call again.

When we commenced business, a window was solicited, a small table, a couple of split-bottom chairs placed in position, the case of instruments unrolled and spread out on the little table, a towel and a tumbler of water completed the arrangements. Now to business: Our patient was seated in the chair next the window, ourself stationed on the right, our left foot placed in chair *number two* behind our patient, the towel spread gracefully over our knee, and our patient's head laid gently thereon, face heavenward, mouth ajar, and we were in for it, our back observing the shape of a hoop. At that time we had never seen a dental chair, only a picture of one in Snell's old work on Dentistry. We did not pick up facts then with the same facility that we do in dental matters now, each dentist as a rule, kept what little he knew to himself, and very little it was too. Once in a while some less cautious than others would let drop an idea which was at once gobbled up like a duck would gobble up a June bug.

*Dental Register*, July, 1873, p. 298, writer speaking of the immunity of animal's teeth from decay, says: "It is stated that the South Sea Islanders have remarkably fine teeth, which they file off occasionally; the latter needs confirmation. At all events it is evident that rapid decay takes place on a surface protected from wear by a deposit of 'tartar,' or by the constant presence of other foreign material. There is much yet to learn of enamel."

Now that teeth decay under "tartar," or where protected by "tartar," I have yet to learn. All dentists recognize the fact that tartar protects teeth from decay wherever deposited; even when it accumulates in a carious portion of a tooth it effectually arrests further decay, and that without any cleaning out, as in dental operations. Where there are great accumulations of tartar, "caries does not progress as rapidly as in the opposite extreme, or where there is no tendency to accumulations of tartar.

*Cosmos*, *August*, 1873.—Under the head of "Dental Operations During Pregnancy," writer on page 407 says: "We throw out the very principles necessary to make bone from our staple bread. The blood not containing this element in sufficient quantity, a demand is made upon the parent's system with the consequence we have already mentioned as affecting teeth. Now the exposed pulp when capped, fails to throw out what it has not at command, and cannot shield itself by a deposit of secondary dentine. Thus it is asserted by surgeons of extensive experience, that fractures unite with much more difficulty during pregnancy; that non-uniting fractures are more numerous, and all efforts at irritation will prove fruitless until after delivery."

Now all the above facts as stated, may be met with in fashionable life, where little physical exercise is taken, more or less decidedly pronounced, depending on many causes too numerous to mention. That the condition of teeth and bones as set forth by the writer, pertains to any great extent in the rural districts, where females perform physical labor as a concomitant of their habits, the facts are wanting to establish, supposing the bread and other foods being just about the same, as there is not much difference now-a-days in bread.

The luxurious female, whether pregnant or not, is more subject to caries and tardy bone healing than the stout robust labor-worn female. The one is all nerves and bad digestion, the other all muscles and good digestion; the one subject to acidity of stomach and dental decay, the other not; the one physics off an over meal, the other works it off and does not suffer from it.

That the blood lacks in lime salts as the writer says during pregnancy, may be so or not, as our data are not sufficient to fully prove the fact, and if the fact be proven beyond doubt, this fact may, and no doubt is dependent on imperfect digestion or assimilation of food, and the amount of lime necessary that may have been taken in with the food, even refined bread carried out again as *debris* unassimilated.

There is in almost all cases lime enough for the small amount needed for fœtal development during a period of nine months, which would not even necessitate any additional amount of food during this term to furnish the needed supply.

That refined flour is out of place for all young people and pregnant woman, I will admit, but for the aged, who is being fossilized by lime in excess in the system, I think fine flour lengthens the chances of longevity, other things equal.

June No., 1873, *Missouri Dental Journal*, p. 212.—Writer treating of vital action in connection with dental caries, says: "In the teeth themselves, exostosis is caused by the irritation of a *dead* and *decomposing pulp*, and at other times absorption of the cementum and dentine of the roots takes place from the same cause."

The writer here regards both processes as *past vital*, though the one is a wasting, and the other a building one, or that of deposition. The writer may be correct in both statements, though to my idea in only one, that of disintegration wasting called absorption. The building process, or exostosis, so far as my own researches go, have led me to think otherwise, from the fact that we have no proof of any such action taking place after the death of the pulp is complete, but may be carried on up to that period, and is chiefly caused by irritation and weakening of vital resistance in the interior and exterior of the fang. After internal death then all building processes necessarily ceases, which by careful observation in a sufficient number of cases will I think satisfy any one who has made this process a special study.

*American Dental Association*, 1873.—One speaker says: "With reference to the action of acids and alkalies upon the teeth in the mouth, I am still of the opinion that the alkalies are the most destructive; they act upon the *pabulum* taking away the base.

The presence of acids does not prove that they are the cause of decay. There is nothing so destructive of animal life as the alkalies."

Can the speaker show a case of caries where there never has been acidity of the mouth; or where there has been uniformly an alkaline condition? I doubt it.

Another speaker says: "Alkalies may destroy the teeth by overcoming vital force, and by causing the elimination of an acid destructive. Alkalies will not destroy a tooth if immersed in them."

In this case the speaker did not explain how alkalies overcome vital force, hence we are still in the woods in relation to how the thing is done. Neither can we understand how the presence of an alkali can eliminate an acid in or out of the body.

Another explaining how alkalies destroy the teeth, says: "These alkalies enter the blood vessels, then coming directly in contact with the tooth substance, through the local circulation, destroy the *pabulum* of which the tooth is formed."

This explains the whole thing, though I must confess I do not exactly understand it yet, there seems to be some obscurity still unexplained, at least n my mind. The subject was ably discussed by other speakers.

Another speaker remarks, "I think the truth will be found between the two theories. Alkalies enter the tooth at the *distal* end of the tubuli and interglobular spaces, and act upon the tissues. Its affinities for the *pabulum* causes it to attack the teeth in passing the interglobular spaces, being attracted by the pond of supply."

This last speaker has got us still further into the wilderness. I do not exactly understand where the distal ends of tubuli are, and from his remarks he seems to entertain the idea that interglobular spaces are always to be found in all teeth, especially when they decay; this is something *new* again. So far I am only able to discover theory instead of demonstrable facts and proofs, which I imagine cannot be given in the premises.

Another speaker having noticed the evil effects of alkalies on the teeth, says: "In the constant use of baking powders and soda, the phosphates are eliminated and the *pabulum* destroyed."

I cannot see the proof in any of the above statements. I do not understand how baking powders can render the secretions acid, so as to take away the phosphates, after such powders have removed the *pabulum*. Other arguments were used but not exactly explanatory and satisfactory. It is rather singular that none of our late society meetings have taken up and discussed *Bumgardner's* new method of pulling teeth. it certainly is the latest novelty in our specialty.

*Care of the Teeth.*—By the Tennessee Dental Association, Nashville, 1873. Under the heading of "Caries of Teeth," I notice the chemical constituents of the teeth as given by Berselius, in 100 parts, viz:

| | |
|---|---|
| Phosphate of Lime, - - - - - - - - | 85.3 |
| Fluate of Lime, - - - - - - - - | 3.2 |
| Carbonate of Lime, - - - - - - - - | 8. |
| Phosphate of Magnesia, - - - - - - | 1.5 |
| Soda and Muriate of Soda, - - - - - - | 1. |
| Animal matter and water, - - - - - - - | 1. |
| Total, | 100 |

Says: "Any one at all familiar with the principles of chemistry, would at once conclude that the above named salts, unless protected by a large amount of animal matter, would be readily acted upon by acids of every kind. Then, too, the low degree of vitality existing in the teeth greatly favors this combination. Acids will not act upon living healthy bone, because the vital force holds in obeyance the force of chemical affinity; but upon dead bone they act with such rapidity that they have taken the place of saw and chisel in many cases where the removal of carious bone is required. In the teeth, however, the vitality is so low that repair never takes place when injury is done to their structure, and they seem to be given up to mechanical and chemical influences."

I give the analysis copied from "Peasley's Human Histology of the Teeth."

DENTINE SOLID SUBSTANCE.

| | |
|---|---|
| Phosphate of Lime, - - - - - - - - | 64.$\frac{1}{3}$ |
| Carbonate of Lime, - - - - - - - - - | 5.$\frac{1}{3}$ |
| Phosphate of Magnesia, Soda and Salt, - - - | 2.$\frac{1}{3}$ |
| Organic Substance, (Osteine,) - - - - - - | 28. |
| Total, | 100.00 |

ENAMEL.

| | |
|---|---|
| Organic Matter, | 3.59 |
| Inorganic Matter, | 96.41 |
| Total, | 100 00 |

CEMENTUM.

| | |
|---|---|
| Organic Matter, (Osteine,) | 32.24 |
| Earthy Matter, | 67.76 |
| Total, | 100.00 |

| | |
|---|---|
| Phosphate of Lime and Fluoride of Calcium, | 58.73 |
| Carbonate of Lime, | 7.22 |
| Phosphate of Magnesia, | .99 |
| Salts, | .82 |
| Cartilage, (Osteine,) | 31.31 |
| Fat, | .93 |
| Total, | 100.00 |

ENAMEL.

| | |
|---|---|
| Phosphate of Lime, some Fluoride of Calcium, | 89.82 |
| Carbonate of Lime, | 4.37 |
| Phosphate Magnesia, | 1.34 |
| Salts, | .88 |
| Cartilage, | 3.39 |
| Fat, | .20 |
| Total, | 100.00 |

Analysis of bones are about the same as that of cementum, 33 organic, and 67 mineral. The analysis in this little pamphlet was by Berzelius, who flourished fifty years ago, and could refer to the enamel alone, and not to the teeth in whole. This committee should have been more particular in giving the chemical equivalents of the teeth in a popular treatise of the kind, as there are learned men in Nashville as well as in other places, that would readily detect the error. This analysis shows only one per cent. in the 100 of animal or organic matter proper, which would teach the community that the teeth were truly dead organs in their mouths, and not live things at all.

The ideas advanced in relation to vital resistance in living bones, and the ready disintegration of dead bones, are in both instances overdrawn ; as dead and detached pieces of bone may and do often remain a long time in many instances without any great amount of disintegration. Many such instances have come under my own observation. As to true vital resistance in bones being so much greater than in teeth, it lacks data to sustain the statement. The teeth in fact are highly endowed with nerves, and from their naked and exposed condition have truly wonderful vital resisting powers, so far superior to any other bones. Bones are covered up and protected from all outside enemies, but of very low vital endowment *per se*.

